# Practical Guidance for the Expanded Implementation and Provision of Bispecific Antibodies for Diffuse Large B-Cell Lymphoma (DLBCL) Across Canada

**DOI:** 10.3390/curroncol32080460

**Published:** 2025-08-15

**Authors:** David MacDonald, Robert Puckrin, Pamela Skrabek, Selay Lam, Jai Jayakar, Isabelle Fleury, Christopher Lemieux, Mélina Boutin, Jacqueline Costello

**Affiliations:** 1 Division of Hematology, The Ottawa Hospital, Ottawa, ON K1H 8L6, Canada; 2 Arthur J.E. Child Comprehensive Cancer Centre, Calgary, AB T2N 5G2, Canada; 3 Department of Medicine, University of Calgary, Calgary, AB T2N 1N4, Canada; 4 Section of Medical Oncology/Hematology, Department of Internal Medicine, Max Rady College of Medicine, University of Manitoba, Winnipeg, MB R3A TR9, Canada; 5 CancerCare Manitoba, Winnipeg, MB R3E 0V9, Canada; 6 Division of Hematology, Department of Medicine, Western University, London Health Sciences Centre, London, ON N6A 5W9, Canada; 7 Division of Hematology, Department of Medicine, Southlake Health, Newmarket, ON L3Y 2P9, Canada; 8 Hematology-Oncology and Cell Therapy University Institute, Hôpital Maisonneuve-Rosemont, Montréal, QC H1T 2M4, Canada; 9 Department of Medicine, Université de Montréal, Montréal, QC H3T 1J4, Canada; 10 Divison of Hemato-Oncology and Transplantation, CHU de Québec-Université, Laval, QC G1V 0E8, Canada; 11 Division of Hemato-Oncology, Charles-Lemoyne Research Center, Greenfield Park, QC J4V 2G9, Canada; 12 Faculté de Médecine et des Sciences de la Santé, Université de Sherbrooke, Sherbrooke, QC J1K 2R1, Canada; 13 Faculty of Medicine, Memorial University of Newfoundland, St. John’s, NL A1B 3V6, Canada; 14 Eastern Health, St. John’s, NL A1B 3V6, Canada

**Keywords:** diffuse large B-cell lymphoma, bispecific antibodies, T-cell engaging therapy, relapsed refractory lymphoma, epcoritamab, glofitamab, odronextamab

## Abstract

(1) Background: Bispecific antibodies (BsAbs) for the treatment of relapsed/refractory diffuse large B-cell lymphoma (R/R DLBCL) can be delivered in ambulatory healthcare settings; however, the safe and effective management of potential side effects, such as cytokine release syndrome (CRS), requires protocolized monitoring and management. (2) Methods: An Expert Working Group (EWG) of nine hematologists from across Canada, with experience in leading BsAb program implementation, combined a review of published literature, a comparison of national/provincial/regional guidance documents and protocols, and their professional experiences to produce an informed framework for BsAb program implementation in various healthcare settings. (3) Results: The EWG supports and recommends the progression of BsAb provision from predominantly inpatient hospital settings to community/ambulatory care settings closer to the patient’s home. A seven-step implementation process is outlined to support the safe and effective establishment of such programs, from establishing leadership, through customization of protocols, to education and execution. Strategies and considerations are offered to overcome potential barriers and empower healthcare professionals who are working to establish or improve BsAb programs across Canada. (4) Conclusions: For patients with R/R DLBCL, the safe and effective provision of BsAbs closer to home is both feasible and preferred. This guidance is intended to support the efficient and effective setup or enhancement of BsAb programs in lymphoma.

## Simple Summary

Bispecific antibodies (BsAbs) are a relatively new treatment option for relapsed/refractory diffuse large B-cell lymphoma (R/R DLBCL). There is an opportunity to provide these medications in the community, with greater convenience for patients, but establishing such programs requires safe and effective administration, monitoring and management. In this paper, an expert working group of nine hematologists from across Canada provides guidance on setting up and enhancing BsAb programs for lymphoma.

## 1. Introduction

Diffuse large B-cell lymphoma (DLBCL) is the most common subtype of aggressive lymphoma [1]. Although many patients are cured by effective front-line treatments (e.g., rituximab plus cyclophosphamide, doxorubicin, vincristine, and prednisone; R-CHOP or chemoimmunotherapy, CIT), morphologic and molecular heterogeneity drive high rates of relapse (20–40%) or treatment refractoriness (10–15%) [1,2,3,4,5]. Patients with high-risk features (e.g., double- or triple-hit genetic rearrangements or central nervous system involvement) may benefit from additional or alternative chemotherapy [5]. Patients with relapsed/refractory (R/R) DLBCL have a growing number of treatment options that provide a chance at cure or prolonged survival [6,7]. These include chimeric antigen receptor T-cell (CAR-T cell) therapy or chemotherapy followed by autologous stem cell transplant (ASCT) [3,6,7,8]. While CAR-T cell therapy has become standard second-line treatment for eligible patients with early relapse, alternative therapies may be indicated or preferred in cases where comorbidities, finances/logistics, or place of residence pose constraints to receipt of CAR-T cell therapy [6]. In addition, the majority of patients (35% and 60–70%, respectively) will relapse following CAR-T cell treatment or chemotherapy followed by ASCT and require subsequent therapies [9,10].

A new class of agents, bispecific antibodies (BsAbs), target both B-cells (via CD20, CD19 or CD22 binding) and T-cells (via CD3 binding) and are commonly referred to as ‘bispecifics’. Following pivotal studies demonstrating manageable safety and durable responses, Health Canada approved, with conditions, two “off-the-shelf” CD3/CD20-targeting BsAbs: epcoritamab and glofitamab [11,12,13,14,15,16], while odronextamab is pending review [17]. Epcoritamab is indicated for the treatment of adult patients with R/R DLBCL not otherwise specified, DLBCL transformed from indolent lymphoma, high-grade B-cell lymphoma (HGBCL), primary mediastinal B-cell lymphoma (PMBCL) or follicular lymphoma Grade 3B (FLG3b) after two or more lines of systemic therapy and who have previously received or are unable to receive CAR-T cell therapy [11]. Glofitamab is indicated for the treatment of adult patients with R/R DLBCL not otherwise specified, DLBCL arising from follicular lymphoma (trFL), or PMBCL, who have received two or more lines of systemic therapy and are ineligible to receive or cannot receive CAR-T cell therapy or have previously received CAR-T cell therapy [12]. Glofitamab, a fixed duration treatment, is given by intravenous infusion up to a maximum of twelve 21-day cycles (13 doses over 36 weeks) or until disease progression or unacceptable toxicity [12]. Epcoritamab, an indefinite duration treatment, is administered by subcutaneous injection once weekly for 3 months, then once every 2 weeks for 6 months (24 doses over the initial 36 weeks), and then once every 4 weeks thereafter until disease progression or unacceptable toxicity [11]. Other therapeutic strategies for patients ineligible for or relapsing following CAR-T cell therapy include polatuzumab-rituximab-bendamustine (funded outside of Québec), tafasitamab-lenalidomide (funded only in Québec), loncastuximab (not funded in Canada), or chemotherapy-based regimens such as rituximab-gemcitabine-oxaliplatin (R-GemOx) [8,18]. Furthermore, approval of the novel combination Glofit-GemOx is anticipated, considering results from the recently reported phase III study STARGLO, where a significant overall survival benefit was demonstrated over R-GemOx in transplant-ineligible patients with R/R DLBCL after one or more previous lines of therapy [19].

Selection and sequencing of therapy for R/R DLBCL continues to evolve in the absence of head-to-head data, with pivotal trial sub-analyses and real-world evidence loosely informing personalized treatment selection and broader funding criteria [8,20,21,22]. However, an optimal treatment sequence has yet to be established. In addition to safety and efficacy data, choosing a BsAb over other therapies may be driven by patient age, comorbidities, ability/willingness to travel to a treatment centre, performance status, preference (route of administration, frequency, duration of treatment, side effects), tumour burden, disease progression kinetics, caregiver support, or accessibility to treatment in the community setting [23,24,25,26,27,28].

By targeting the immune system, T-cell engaging therapies can trigger cytokine release syndrome (CRS) and neurotoxicity. With CAR-T cell therapy, rates of any grade/grade ≥ 3 CRS have been 49–95%/1–24%, and any grade/grade ≥ 3 neurologic toxic effects from 12 to 60% and 3 to 50% have been observed in trial settings [29]. Common toxicities of CD3/CD20-targeting BsAbs reflect their mechanism of action, stimulating T-cells and inhibiting B-cells, and include CRS, cytopenia, and infections [6,11,12]. In the pivotal phase I/II trials for BsAbs as third-line or later treatment for DLBCL, the proportion of patients on epcoritamab and glofitamab, respectively, experiencing any grade/grade ≥ 3 CRS events was 51%/3% and 64%/4%, and immune effector cell-associated neurotoxicity syndrome (ICANS) was 6%/1% and 8%/3%, highlighting a generally lower incidence and severity of these toxicities with BsAbs compared to CAR-T cell therapy [3,13,14,30].

CD3/CD20 BsAb administration protocols are designed to mitigate CRS/ICANS risk through pretreatment with corticosteroids, antipyretics, and antihistamines; step-up dosing (SUD) of the BsAb; subcutaneous (SC) or slower intravenous (IV) administration; and, for glofitamab, administration of a preceding dose of obinutuzumab to deplete circulating B-cells [3,11,12]. CRS events are most likely to occur in the first cycle of treatment, during SUD, with no grade ≥ 3 CRS events reported after cycle 1 for either BsAb in pivotal studies [13,14]. Furthermore, upon the third full dose of epcoritamab or glofitamab, the incidence of grade 1 or 2 CRS dropped to 3% and 2% for each therapy, respectively [13,14]. Optimization of corticosteroid pre-treatment (i.e., exclusive use of dexamethasone versus any corticosteroid) and hydration during SUD further reduced the incidence and severity of CRS; for glofitamab, CRS events were reduced by 34%, with all grade/grade ≥ 3 CRS experienced by 48.5%/3.0% in the ‘dexamethasone only’ cohort versus 73.2%/4.5% in the ‘any corticosteroid’ cohort; and for epcoritamab, the incidence of all grade/grade ≥ 3 CRS was 49.7%/2.5% in the ‘any corticosteroid’ cohort versus 17%/0% following optimization protocols [31,32].

Numerous guidelines exist to support the effective management of these toxicities [33,34]. Due to the increased risk of CRS and ICANS, CAR-T cell recipients are primarily managed in inpatient settings at a small number of specialized academic centres in Canada [35]. In contrast, the favourable safety profile of CD3/CD20 BsAbs do not mandate hospitalization, allowing for administration and monitoring in the outpatient and community hospital settings [11,12,36]. However, given the risk of potentially serious adverse events (AEs), particularly CRS, following BsAb administration, planning for close monitoring and access to early, specialized and multidisciplinary care is important [34].

Grading for CRS and ICANS is standardized, and patients should be monitored to identify symptoms, including fever, malaise, hypoxia, and hypotension, as early as possible [33]. In the glofitamab pivotal study, the median time to onset of the first CRS event from the cycle 1, day 8 dose was 13.6 h (range, 6.2 to 51.8), with the event lasting a median of 30.6 h (range, 0.5 to 316.7) [13]. This is slightly prolonged with subcutaneous administration of epcoritamab, with a median time to CRS onset of 20 h following the first full dose (cycle 1, day 15) and a median time to resolution of 48 h [14,37].

Management of CRS has been optimized through experience with CAR-T cell therapy and was recently adapted for BsAbs in DLBCL and multiple myeloma (MM) [34,38,39]. Of operational importance, CRS management may require the use of anti-cytokine therapy, such as tocilizumab, which needs to be proactively stocked by hospital pharmacies administering BsAbs or managing CRS [34]. Optimization with dexamethasone prophylaxis in patients receiving glofitamab reduced intensive care unit (ICU) admission for CRS management (5.4% in those treated with any corticosteroid vs. 3.0% in those treated with dexamethasone only) [31]. Similar data is not yet available for epcoritamab. This means that fewer than 1 in 20 treated patients is expected to require ICU support. Special consideration should be given to rechallenge in cases of higher-grade CRS and ICANS, as these therapies should be permanently discontinued in the event of grade 4 toxicity, and risk/benefit carefully re-evaluated following grade 3 events [11,12]. Furthermore, after a prolonged treatment interruption, the initial ramp-up schedule for risk mitigation should be repeated [11,12]. With appropriate intervention, recurrence following re-treatment is rare [13,14].

Current availability and future growth in demand reinforce the importance of establishing accessible, safe, and effective BsAb administration protocols [3,19]. The reimbursement applications for epcoritamab and glofitamab highlight the unmet needs and poor prognosis of patients with R/R DLBCL who are unable to receive or have relapsed after curative therapy [40,41]. These reports note geographic (travel vulnerability) and healthcare system barriers that prevent patients from receiving other treatments and underscore the need for community provision of BsAbs closer to home across Canada’s vast geography and within its varied health systems [40,41,42]. Furthermore, BsAbs continue to be studied in earlier lines of treatment and in combination with other agents for DLBCL and various malignancies, so the need for safe and accessible BsAb programs is expected to grow [25,34,43,44].

Considering the concentrated experience of BsAb provision in Canadian academic centres, an Expert Working Group (EWG) was assembled to share perspectives and best practices related to the potential operationalization and expansion of BsAbs programs into community settings across the country.

## 2. Materials and Methods

### 2.1. Expert Working Group (EWG) Formation

In early 2025, an EWG was assembled with the intent to bring together healthcare professionals (HCPs) with experience in treating R/R DLBCL with BsAbs and leading the establishment of a program for BsAbs provision at their centre. Pan-Canadian representation was a priority, and the inclusion of various health system experiences (i.e., academic and community) was intended. Following the email invitation, nine hematologists from nine treatment centres (5 academic/CAR-T, 3 academic, and 1 community) formed the final EWG. At inception, the EWG had a collective experience of over 100 patients with DLBCL treated with BsAbs (epcoritamab or glofitamab).

### 2.2. Literature Review and EWG Alignment

An internet search (including PubMed, Google and ASH abstracts) was conducted to identify publicly available data, guidance, or resources related to epcoritamab or glofitamab in DLBCL and included search terms: ‘epcoritamab’, ‘glofitamab’, ‘bispecific’, ‘bispecific antibody’, ‘T-cell engaging antibody’, ‘DLBCL’, ‘CRS’, ‘ICANS’, and the following provinces/provincial cancer agencies: ‘British Columbia’/‘BC Cancer (BCCA)’, ‘Alberta’/‘Cancer Care Alberta (CCA)’, ‘Saskatchewan’/‘Saskatchewan Cancer Agency’, ‘Manitoba’/‘CancerCare Manitoba (CCMB)’, ‘Ontario’/‘Cancer Care Ontario (CCO)’, ‘Quebec’/‘Institut National d’Excellence en Santé et en Services Sociaux (INESSS)’, ‘New Brunswick’/‘New Brunswick Cancer Network’, ‘Nova Scotia’/‘Nova Scotia Cancer Care Program’, or ‘Newfoundland and Labrador’.

EWG members completed a pre-meeting survey (Table A1) to share their experiences related to BsAb program implementation in DLBCL. In this paper a ‘BsAb program’ is defined as the provision (administration, monitoring, and care) of BsAbs for the treatment of R/R DLBCL. In February 2025, the EWG met virtually to discuss responses to the survey in the context of current related literature. The EWG aligned on a BsAb implementation framework and agreed the publication should share the range of experience across Canada, with recommendations that could be considered to apply to the varied systems and unique capabilities and needs of each centre.

## 3. Results and Discussion

With a pressure to expand BsAb programs into the community, it is critical to consider the Reflecting on the establishment of the BsAb programs in their centres, the authors formulated a ‘Steps to implementing a BsAb program’ guidance (see Table 1).

### 3.1. Step 1. Establish Physician Leadership for Implementation

Appointing a physician lead, or assuming a leadership role in developing a BsAb program, is the definitive starting point to implementation. The key to success in this step is having a motivating goal and applying strong leadership skills that will support the development of a team in which collaboration, communication, and outcomes are prioritized. Sharing leadership might be of benefit, particularly in community settings where another specialty (e.g., medical oncology) has interest in or a need to offer BsAbs. Early engagement of other leaders from nursing, pharmacy, and administration can also be helpful.

For most of the EWG, the motivation to lead the BsAb program implementation was most often a patient case (current or anticipated) and the knowledge that establishing a program in their centre would be necessary in order to address unmet needs in this patient population. Both the missed opportunities, where patients were unable to receive BsAb therapy, and the cases of success bolstered the need for programs. As experience with BsAbs in DLBCL grows, motivation has expanded to include a desire to support patient quality of life and care through program optimizations and expansion of offerings closer to the patients’ homes [36,45]. In a community hospital setting, BsAb program leadership will likely fall to the treating physician, with a patient needing BsAb treatment closer to home.

### 3.2. Step 2. Explore Potential Care Pathways

As the use of and experience with BsAbs in DLBCL grows, the strain on central cancer centres and patients has become more apparent [36]. In Canada, 18% of the population resides outside of urban centres, making travel vulnerability related to healthcare delivery a real concern, particularly for specialized services [36,46]. The northern provinces and territories, Atlantic provinces, Saskatchewan, and Manitoba have the highest share of people living in rural areas (35–55%, 40–54%, 32% and >25%, respectively) [46].

When leaders are faced with the desire or need to expand the provision of BsAbs beyond core academic centres and into a community hospital setting, consideration must be given to the current health system, its services and capacities, and how those can be utilized or adapted to accommodate these novel treatment offerings. In the case of BsAbs for R/R DLBCL, consideration must be given to both patient and system capacity to manage treatment, from assessment/preparation to administration, monitoring, and management of AEs, including CRS (Table 2). Academic members of the EWG relayed hurdles to implementation that included securing clinic space/resources, knowledge translation from CAR-T-focused teams on CRS management, securing formulary funding of tocilizumab, and coordination between various specialties and centres. Those experienced in the setup of outpatient programs in the community faced the need to increase awareness of unique BsAb toxicities (e.g., CRS and ICANs) and ensure appropriate support for after-hours management of patients with unscheduled complications by engaging stakeholders, such as the ER, general internal medicine, hospitalists, and critical care. For all centres, a commitment to developing customized resources and partner education was central to success.

**Figure 1:**
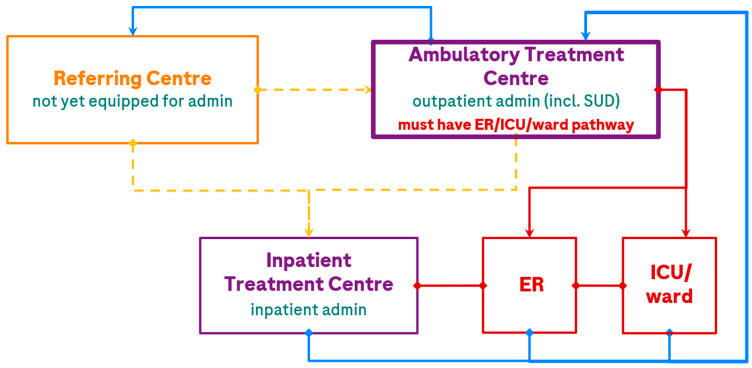
Potential patient care pathways in the treatment of DLBCL with a BsAb. The treatment centre is the location where patients receive BsAb treatment; the ER and ICU may or may not be at the same location. Transitions of patient care are depicted by <yellow> for referral, <red> for escalations of care (e.g., AE management), and <blue> for de-escalations of care (e.g., maintenance dosing or repatriation). The dashed line indicates a current referral pathway that will gradually be utilized less as Referral and Ambulatory Treatment Centres gain experience and capacity for SUD administration. admin, administration; ER, emergency room; ICU, intensive care unit; incl., including; SUD, step-up dosing.

Anticipated capabilities (Table 2) will shape the care path and transitions within it. In Canada, CCO and INESSS, and in the USA, the Association of Community Cancer Centers (ACCC) have published organizational readiness recommendations for BsAb program implementation, which outline elements that should be in place at facilities to support the delivery of high-quality, consistent care [36,47,48]. Considering delivery of care for patients receiving a BsAb for R/R DLBCL in Canada, and the requirements in these guidances, the EWG outlined potential care pathways (Figure 1). Here, transitions of patient care include referrals, escalations of care (such as SUD or AE management), and de-escalations of care (such as maintenance dosing following SUD).

**Figure 2:**
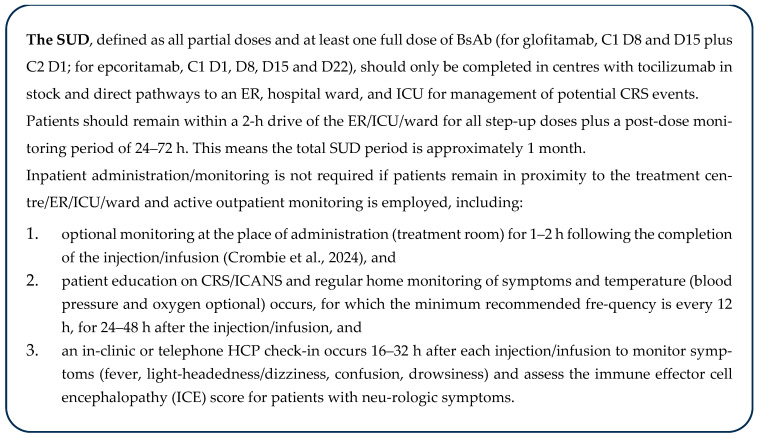
Recommendations for outpatient administration and monitoring for SUD [34]. C, cycle; CRS, cytokine release syndrome; D, day; ER, emergency room; HCP, healthcare professional; ICANS, immune effector cell-associated neurotoxicity syndrome; ICE, immune effector cell encephalopathy; ICU, intensive care unit; NL, Newfoundland and Labrador; SUD, step-up dosing.

The EWG felt that ambulatory treatment closest to home, with appropriate protocols in place, should be the target patient care pathway for future BsAb provision. Where ambulatory treatment centres have clear pathways to ER, inpatient ward beds, and ICU support within approximately 2 h of a patient’s home, all dosing can be provided outpatient if the centre meets organizational readiness criteria (including provincial requirements and recommendations in Figure 2), although some patients may optionally undergo brief hospital admission for CRS monitoring (e.g., after the high-risk dose during SUD). For patients > 2 h from an ambulatory treatment centre, or if the ambulatory treatment centre has only distant access to an ICU (e.g., patients can be stabilized in a local ER but would require flight transfer to an ICU), patients should be referred to a fully equipped treatment centre, which would, at least, complete SUD. Following this, all patients could be de-escalated back to the local ambulatory treatment centre or referring centre for maintenance treatment. Other escalations of care are described in the monitoring section below.

As an example, in Lethbridge, Alberta, the Jack Ady Cancer Centre is now equipped to offer BsAbs from the start of therapy (SUD) with the support of the ER/ICU/inpatient ward at the adjoining Chinook Regional Hospital, to reduce strain on the Arthur J.E. Child Cancer Centre in Calgary and support patients closer to home (saving travel expenses and burden associated with a >2 h drive to or overnight stays in Calgary).

### 3.3. Step 3. Identify and Engage Multidisciplinary Partner Leads

**Figure 3:**
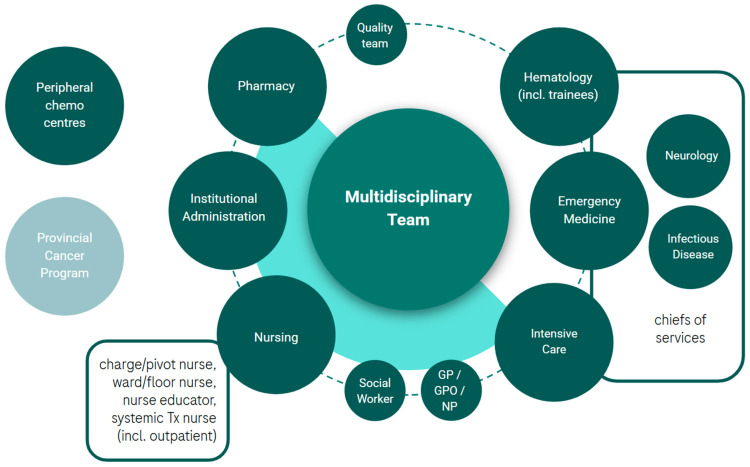
Potential multidisciplinary partners involved in the implementation of a BsAbs program in R/R DLBCL. chemo, chemotherapy; GP, general practitioner; GPO, general practitioner of oncology; incl., including; NP, nurse practitioner; Tx, treatment.

Multidisciplinary engagement was identified as necessary to the successful development of a BsAbs program. While the list of potential partners was large (see Figure 3) it was deemed important to start with a small team of leads for initial support. Project leads are encouraged to review Figure 3 and consider partners who would be instrumental to the success of the program, considering capability requirements (Table 2), their local health system dynamics (Figure 1), the partner’s role/authority, and their influence. Key roles that were common in the establishment of programs at the EWG member’s institutions included a(n): ER physician, ICU physician, neurologist, pharmacist, systemic treatment and inpatient ward nursing management, a charge/pivot nurse, and an institutional administrator. Similarly to the establishment of the project lead role, multidisciplinary lead engagement can benefit from leveraging a common goal (e.g., leading implementation or patient benefit), which may be integrated with general education around BsAbs and the unmet need for patients with R/R DLBCL. To this end, patient advocacy may also support program implementation.

### 3.4. Step 4. Define Patient Care Pathways and Unique Centre/Partner Capabilities

In this step, the project and multidisciplinary leads align on the ideal patient care path considering their health system (Figure 1) and capabilities (Table 2 and Figure 2). Example care paths currently operating in Canada are shared in Table A2. The leads now also consider the engagement of additional multidisciplinary partners (Figure 3) in the development of resources and consideration of strategies (Step 5) to address organizational readiness and overcome barriers. This engagement of a broad, multidisciplinary team will enrich the content created and provide optimal, hands-on learning for those involved. This is a critical step in the setup of a program as it integrates various perspectives and co-operatively builds solutions that address barriers across disciplines. The respect for partner capacities, preferences, and experience can build trust amongst the team and foster co-operative endorsement of the project across disciplines.

### 3.5. Step 5. Create or Customize Existing Resources and Strategies to Address Barriers and Support Implementation

#### 3.5.1. Preparation

##### Referral

The EWG agreed that the decision to initiate BsAb treatment and SUD should be the responsibility of the treatment centre conducting the SUD (not the referring centre). To shorten time to treatment and avoid unnecessary travel to tertiary centres, referring centres may reach consensus on clinical indication based on tumour board discussion and select patients eligible for referral. Expectations for de-escalation to ambulatory administration or repatriation to the referring centre should be made clear before treatment initiation. Treatment and referring centres should also delineate roles and responsibilities related to patient education or assessments prior to the transfer of the patient’s care to the treatment centre.

Existing referral forms and pathways, i.e., standard cancer program forms [49], electronic medical records (EMR) forms [50], or ASCT templates [51] may be used or adapted for BsAb referral [35]. The EWG noted no special requirements for BsAb referral beyond the standard laboratory assessments, imaging, pathology reports, and medical and treatment history typically shared [52,53]. In some provinces, EMRs are shared between all institutions, allowing for full transparency. INESSS guidance on BsAb deployment suggests utilization of telehealth technology for virtual initial consultations [36].

##### Patient Assessments

In addition to clinical assessment for treatment eligibility, patient preferences and goals for treatment should be understood. Social constraints or supports should be explored by the treatment team, including financial burden, travel vulnerability, cognitive ability, health literacy, ability to self-monitor, degree/capability of caregiver support, and required supplies (e.g., thermometer) [34,54]. Rural patients may require help with travel or lodging, in which case a social worker could be engaged or supports offered through the Canadian Cancer Society, Leukemia & Lymphoma Society travel subsidy, or health ministry or institutional travel policies could be explored [36,55].

Patients should also be assessed prior to treatment for the risk of developing CRS. To assess risk, EWG members use clinical judgement/Gestalt, informed by known risk factors such as tumour burden, elevated lactate dehydrogenase (LDH), advanced stage, extranodal disease, peripheral blood or bone marrow involvement, and patient characteristics (older age, and comorbidities) [56]. Although not practical for everyday clinical use, prediction models support the use of certain variables in predicting the risk of CRS. For glofitamab candidates, the weighted inclusion of age > 64 yrs, LDH > 280 U/L, white blood cell (WBC) > 4.5 × 10^9^ cells/L, Ann Arbor stage IIII/IV disease, and sum of the product of the perpendicular diameters ≥ 3000 mm^3^ were found to be predictors of CRS events [57]. For patients who may receive epcoritamab, prior CAR-T cell therapy, extranodal disease, and total metabolic tumour volume were predictive of grade ≥ 2 CRS [58]. Patients deemed at higher risk of CRS events may require consideration for inpatient monitoring for certain high-risk step-up doses to optimize the management of emergent events.

##### Patient Education

Crombie et al. (2024) detail ideal requirements for patient education, which the EWG endorse [34]. A number of resources from the public domain exist and can be adapted to each centre, including information on BsAbs [59,60,61], CRS/ICANS information sheets [62,63], self-monitoring guidance [64], and wallet cards [65,66,67] or triage letters [68,69] to carry for sharing with ER staff or other HCPs [34,35]. The EWG notes that information can be overwhelming for patients and it is important to provide simple and clear education at multiple time points (i.e., before the treatment decision, before premedication administration, and with each treatment).

##### After-Hours Coverage in Community Settings

At the time of this writing, many Canadian community centres have not yet implemented administration of BsAbs. Although provider education is an important barrier here, this can be easily addressed through appropriate education materials and presentations. A more pressing barrier for implementation in community settings is the lack of physician human resources within the hematology or cancer programs to provide timely after-hours or unscheduled care for patients presenting with potential CRS and/or ICANS. This can create significant resistance to implementation from overburdened providers who may already be covering a range of disease sites on call. Engaging stakeholders outside the cancer program, such as ER, general internal medicine, hospitalists, and critical care, to assess and manage patients with these toxicities (using protocols and order sets, with support being available from the hematologist on call, if available), has proven to be a successful model in the few community centres that have successfully implemented SUD and beyond for BsAbs. However, in cases where adequate after-hours support would not be available, referral for SUD is recommended.

#### 3.5.2. Administration

##### Protocols

Administration protocols should be aligned with product prescribing information and supplemented with published experience where applicable [11,12]. The EWG supports the inclusion of optimization practices (Table 3) in administration protocols, including strong preference for dexamethasone as the steroid premedication of choice to support reduced frequency and severity of CRS [13,31,32]. There was also agreement among the EWG that protocols be easy to follow, highlighting Cancer Care Manitoba’s [70,71], for example, which were developed in close collaboration with key nursing partners. The EWG encourages the inclusion of administration instructions for dose interruptions or prior AEs, in line with the product monographs [11,12].

While prophylactic tocilizumab has been used in MM for BsAb-related CRS prevention [73,74,75,76,77], data is not currently available to support its use in lymphoma [45]. Such use in lymphoma is considered experimental and not recommended as standard practice by the EWG. Prophylactic tocilizumab use may also be met with funding restrictions by some provincial cancer agencies. To support the collection of prospective data on this practice, one member of the EWG is compiling a case series detailing prophylactic tocilizumab use prior to BsAb administration in R/R DLBCL.

#### 3.5.3. Monitoring

The EWG acknowledges that instructions for monitoring patients during and after BsAb administration are not finely detailed within the product monographs [11,12] and interpretation and implementation vary widely between provincial/centre protocols [21,22,36,45,70,71,78,79,80]. The EWG encourages the development of detailed and clear protocols that integrate product monograph recommendations, clinical experience, and patient transitions of care (i.e., inpatient to outpatient to home monitoring) relevant to the local health system in question.

The EWG believes that the recommendations in Figure 2 support the safe administration of BsAbs, are in alignment with each product’s prescribing information, and enable relief of inpatient administration while supporting patient quality of life and enabling community provision more broadly across Canada, where feasible and safe:

An example of Figure 3 recommendations combined with the patient care pathway (Figure 1) is: A patient from The Pas in MB, an ~6 h drive from Winnipeg, stays in proximity to Cancer Care Manitoba for 3 days following each step-up dose. Following step-up dosing, the patient returns home and receives maintenance doses at the local Community Cancer Program site.

The boxed recommendation (Figure 2) should be extended for subsequent doses in patients who experience a CRS/ICANS event during SUD, until one full dose has been administered without CRS/ICANS, or for the repetition of SUD following treatment interruptions (defined in the prescribing information). Inpatient administration should be considered for patients at higher risk of CRS events (see risk assessment), particularly after specific high-risk doses (e.g., C1D15 for epcoritamab or C1D8 for glofitamab), in those who have experienced a CRS event with prior administration, and for patients lacking support or the ability to effectively monitor or seek escalated care.

The EWG endorses the outpatient administration of BsAb maintenance dosing at any treatment centre once SUD has been safely completed. These centres may consider proactively stocking tocilizumab; however, given the low risk of recurrent CRS post-SUD and high likelihood that any recurrent CRS would be grade 1, the use of tocilizumab is usually not required [34].

While the above recommendations do not reflect current practice at all sites across Canada, they are both aspirational and practical, in support of the expanded implementation of BsAbs in Canada.

#### 3.5.4. CRS/ICANS Management

##### Protocols/Standing Orders

The EWG endorses general CRS/ICANS management guidelines [33], including use of the CARTOX app (MD Anderson Cancer Center), product monograph recommendations [11,12], and the guidance provided by Crombie et al. (2024) adapted specifically for lymphoma BsAb-induced CRS/ICANS [34]. These have been largely reflected in provincial protocols for CRS/ICANS management [21,22,45,70,71,78,81,82,83]. However, the EWG emphasizes the importance of maintaining awareness of and exploring other aetiologies in the face of suspected CRS/ICANS (e.g., infection, cardiovascular events, central nervous system lymphoma, rapidly progressing disease, etc.) [34]. The EWG supports potential at home management of grade 1 CRS in appropriately fit patients who have caregiver support, but not for grade ≥ 2 CRS or grade 1 ICANS, for which management, including ICE score assessment, is recommended in clinic/ER/hospital ward. Some members of the EWG provide patients with prescriptions for oral acetaminophen and dexamethasone to have at home and take as directed in the event of suspected grade 1 CRS, with directions including clear instructions on follow-up steps. Finally, the EWG encourages the use of standing orders (examples: BCCA [84,85]) to enable the prompt and standardized management of CRS/ICANS.

##### Triage

The EWG recognizes the very real capacity constraints of emergency rooms (ERs) and shortage of beds in hospitals across the country [86], as well as the potential risk this poses to timely CRS/ICANS management. In addition, limited hematologist staffing may preclude on-call support, necessitating a reliance on ER assessment after hours. To this end, the EWG offers a number of triage solutions to ensure timely and efficient ER attention, or strategies to bypass the ER where possible.

Patient tools for triage (i.e., wallet card and letter) can be employed to support early and effective management of CRS/ICANS events in the outpatient setting. The EWG also recommends, where appropriate, the use of EMR flags and/or inclusion of management protocols in the patient file.

Further, the on-call physician or triage nurse made aware of a potential CRS event can proactively alert the local ER of the patient’s anticipated arrival and share relevant protocols/orders for management.

At Southlake Health, a community hospital, and many academic institutions, the ER is equipped with dexamethasone and tocilizumab, and protocols and order sets have been developed by the cancer program in conjunction with ER stakeholders, to facilitate rapid assessment for patients presenting with potential CRS. The aim is to give interventions within 1 h of presentation, akin to antibiotic administration time targets in febrile neutropenia.

Some centres, such as the Ottawa Hospital, are now employing “flex beds”, where patients receiving outpatient administration of BsAbs remain outpatients but are “admitted” to a ward bed, which is reserved for 48 h following each SUD. This model provides the patient with direct entry to a unit/team that is aware of the patient and is trained in BsAbs AE management, allowing the patient to avoid wait times and potentially unfamiliar staff in the emergency department. Another initiative under consideration to alleviate bed space dedicated to BsAb patients are “Virtual Medicine Wards” or “Hospital-at-Home Programs” [87]. This requires, among other things, a well-selected and co-operative patient, the presence of a 24/7 caregiver, 24/7 virtual access to the nursing team, coordination with home pharmacy delivery and nursing services for blood tests, and a trained physician on call to assess patients for at-home management or ward admission in case of CRS/ICANS. Treatment centres may also equip themselves to manage low grade CRS/ICANs by procuring the necessary equipment and drugs for management, alleviating strain on ERs during clinic hours. This process is facilitated in Manitoba (CancerCare Manitoba MacCharles) and in Québec (Maisonneuve-Rosemont Hospital), for instance, through a centralized urgent cancer care help line trained to direct patients to the outpatient clinic or ER according to symptom severity. At London Health Sciences and Maisonneuve-Rosemont Hospital, patients are directed to a rapid assessment clinic during clinic hours. Triage coverage should support staff training on BsAbs and clear algorithms for escalation of care.

#### 3.5.5. Managing Other AEs

The EWG endorse recommendations from the respective product monographs and Crombie et al. (2024) for the management of non-CRS AEs (i.e., ICANS, tumour flare reaction, cytopenias, hypogammaglobulinemia, infections, and tumour lysis syndrome) [11,12,34].

#### 3.5.6. Longterm Care

##### Repatriation

As noted for referral, the EWG agreed that expectations for de-escalation to ambulatory administration or repatriation to the referring centre should be made clear before treatment initiation. Treatment centres should communicate the transfer of care and highlight ongoing risks (e.g., rare late CRS events, infections), based on the most recent data [15,16,88], to monitor upon return to the referring centre. Recommendations regarding management of late complications (e.g., cytopenias, hypogammaglobulinemia) should be provided by the treatment centre. In the event of a delayed complication, the treating centre should establish mechanisms to provide clinical support to the referring centre whenever deemed necessary as needed. For patients with a sustained response to glofitamab, recovery of B-cells and immunoglobulins starts around 18 months following the end of fixed-duration treatment [89]. It is unknown how this impacts rates of infection over time. For epcoritamab, a sustained decrease in immunoglobulin G levels of ~20% was seen throughout treatment, with infection rates remaining stable over time [88]. Therefore, ongoing monitoring and vigilance are recommended upon repatriation of patients treated with BsAbs.

### 3.6. Step 6. Engage and Educate the Broader Multi-Disciplinary Team

The EWG recommends the following tactics to support education of all program participants:

1. Engagement of multidisciplinary partners in the development of resources (Step 4/5).
2. Storing resources and educational materials centrally, such as in a website, shared drive or Microsoft Teams group that is available system-wide, for easy access/reference.
3. Designated support (such as access to project leads or central cancer centre experts) for ongoing consultation.
4. Multidisciplinary and intra-specialty education (pharmacist to pharmacist or nurse to nurse) is ideal. This has been the experience of all EWG Québec leads, as they engage staff at ambulatory treatment centres.
5. Case sharing and practical, case-based application of algorithms can improve knowledge translation.

### 3.7. Step 7. Ensure Periodic Review/Update of Processes and Education to Support Program Optimization

An essential step in process improvement, this final task is an ongoing one. Changes in personnel, health system dynamics, and product knowledge/experience will necessitate the periodic update of resources, tactics, and education.

The EWG noted the following best practices for program optimization and improvement:

1. Where applicable, engaging quality teams within a centre for ongoing review, maintenance, and improvement of the BsAbs program.
2. Recording educational presentations for easy reference by new hires or those needing to refresh their knowledge on the topic. Eastern Health is among many hospitals supporting their staff in this way.
3. Involving medical trainees in the ongoing education of other learners. This is a common practice at London Health Sciences to support education and patient care in a high-turnover, rotational, teaching hospital environment.
4. Regularly updating resources and educational materials to reflect the growing body of evidence and experience with BsAbs in DLBCL (e.g., steroid/fluid optimization, [31,32] immune cell recovery and impact on infection risk [88,89], real world evidence, etc.).
5. Considering the use of BsAbs in other diseases (i.e., solid tumours, MM, etc.) and where processes/resources should be customized by, or simplified across, disease/product.
6. Utilization of language translation services, particularly for patient education.

Most EWG members found that after setup (at their centre or in supporting peripheral ambulatory treatment centres), BsAb program implementation was smooth, owing to the low frequency of severe CRS events and protocolized management.

## 4. Conclusions

With the approval and funding of novel BsAbs, patients with R/R DLBCL have an opportunity to receive life-extending treatment. Furthermore, these therapies offer the opportunity for treatment closer to home, with the establishment of ambulatory treatment centres equipped for outpatient administration. By combining a stepwise approach to implementation with the evidence-based and practical guidance outlined here, Canadian centres can move toward meeting unmet patient needs within their unique health systems and prepare for the expanded use of BsAbs in oncology.

## Figures and Tables

**Table 1:** Steps to implementing a BsAb program.

1.	Establish physician lead for implementation.			
2.	Explore potential care pathways considering current centre/regional/patient barriers to implementation.	3.	Identify and engage multidisciplinary partner leads (e.g., nursing, pharmacy, ER, ICU, neurology).	
4.	Define patient care pathways, including referral and transitions of care (escalation/de-escalation) processes, based on unique centre/partner capabilities.		6.	Engage and educate the broader multidisciplinary team.
5.	Customize existing resources (e.g., protocols, order sets) for treatment administration and toxicity management. Consider and apply strategies to address centre/regional/patient barriers in support of implementation.			
7.	Ensure periodic review/update of education and processes to support program optimization.			

**Table 2:** Key considerations and capabilities for BsAb program implementation in DLBCL.

**Preparation**
Centre capacity - administration (chairs, human resources) - AE management (ER, inpatient ward beds, ICU, human resources) Team training Patient evaluation - AE risk - capabilities/support - distance/accessibility to facilities Drug access and logistics - drug funded - drug ordered Patient referral Patient education
**Administration**
Scheduling/logistics Premedication(s) protocols (in-/outpatient) Step-up dosing protocols (in-/outpatient) Maintenance dosing protocols (in-/outpatient)
**Monitoring**
Inpatient protocols Outpatient clinic protocols Outpatient home protocols
**CRS/AE Management**
Drugs for CRS management are funded and on hand (e.g., tocilizumab for bispecific-induced CRS, not just CAR-T cell-induced) ER/ICU/ward access Multidisciplinary team training and experience in diagnosing/managing CRS Comprehensive CRS management protocol After-hours/on-call support and triage Other AE management
**Long-term care**
De-escalation of care once CRS risk is low Repatriation after completion of SUD Other medical management

AE, adverse event; CAR-T cell, chimeric antigen receptor T cell; CRS, cytokine release syndrome; ER, emergency room; ICU, intensive care unit; SUD, step-up dosing.

**Table 3:** Summary of administration-related optimization recommendations to supplement the prescribing information for epcoritamab and glofitamab.

Premedication/Prophylactic Measure	Epcoritamab	Glofitamab
hydration	epcoritamab should be administered to adequately hydrated patients [11] strong recommendation for C1 * [32]	glofitamab should be administered to well-hydrated patients [12]
antihypertensive medications	strongly recommended to hold for 24 h prior to epcoritamab SC administrations in C1 * [32]	EWG recommends holding for 24 h prior to obinutuzumab/glofitamab administrations in C1
obinutuzumab		1000 mg single dose pretreatment (C1, D1) ^†^ [12] See obinutuzumab prescribing information for pretreatment [72]
corticosteroids	C1, dexamethasone 15 mg * ^‡^ 30–120 min prior to and for 3 days following each administration of epcoritamab (i.e., C1 D1, D8, D15, and D22 and 15 mg as prophylaxis on C1 D2–4, D9–11, D16–18, and D23–25) [11,32] continued, as above, until no grade 2–4 CRS with prior dose [11]	dexamethasone 20 mg IV * administered as a single dose 60 min prior to administration of obinutuzumab and glofitamab administration during C1–3 [12,13,31] continued, as above, until no CRS with prior dose [12]
diphenhydramine and acetaminophen	diphenhydramine (50 mg oral or IV) ^§^ or equivalent and acetaminophen (650–1000 mg oral) 30–120 min prior to the administration of epcoritamab on C1 D1, D8, D15, and D22 [11,32]	antihistamine, such as IV or oral diphenhydramine (50–100 mg) ^§^ and oral acetaminophen or paracetamol (500–1000 mg) administered at least 30 min prior to each glofitamab infusion [12]

* evidence-based optimization of prescribing information. ^†^ this single dose follows the pivotal DLBCL clinical trials and prescribing information for glofitamab. Splitting the obinutuzumab dose into two administrations (100 mg/900 mg) is recommended in chronic lymphocytic leukemia, where circulating disease results in higher risk for immune related reactions and dose splitting was found to decrease this risk. ^‡^ practically, this can be 4 × 4 mg tablets (i.e., 16 mg). ^§^ EWG encourages the use of antihistamines without CNS-depressant effects. C, cycle; D, day; IV, intravenous; min, minutes; SC, subcutaneous.

## Abbreviations

The following abbreviations are used in this manuscript:

AE	adverse event
Ab	antibody
ACCC	Association of Community Cancer Centers
admin	administration
ASCT	autologous stem cell transplant
BCCA	British Columbia Cancer Agency
BsAb	bispecific antibody
C	cycle
CAR-T cell	chimeric antigen receptor T cell
CCMB	Cancer Care Manitoba-
CCO	Cancer Care Ontario
CIT	chemoimmunotherapy
CRS	cytokine release syndrome
D	day
DLBCL	diffuse large B-cell lymphoma
ER	emergency room
EMR	electronic medical record
EWG	expert working group
GP	general practitioner
h	hours
HCP	healthcare professional
ICANS	immune effector cell-associated neurotoxicity syndrome
ICE	immune effector cell encephalopathy
ICU	intensive care unit
incl	including
INESSS	Institut National d’Excellence en Santé et en Services Sociaux
IV	intravenous
LDH	lactate dehydrogenase
min	minutes
MM	multiple myeloma
NOS	not otherwise specified
R-CHOP	rituximab plus cyclophosphamide, doxorubicin, vincristine, prednisone
R/R	relapse/refractory
SC	subcutaneous
SUD	step-up dosing
Tx	treatment
WBC	white blood cell

## Appendix A

### Appendix A.1

**Table A1:** Pre-Meeting Survey Questions.

1. Please provide the name of your institution so that your responses may be linked.
2. How would you describe your institution? (e.g., academic, community, hospital, etc.)
3. How many DLBCL patients have you treated with epcoritamab? (please enter a numbers only)
4. How many DLBCL patients have you treated with glofitamab? (please enter a numbers only)
5. What has been your role (s) at this institution with respect to bispecific implementation for DLBCL?
6. Which stakeholders did you need to engage to support implementation? Who makes up the multidisciplinary team?
7. How long did it take to establish the bispecifics program at your institution?
8. In terms of education, who (which services) needed information? What information did they need and how has that need been filled?
9. How are patients referred to your centre? What information/support do you request from the referring centre? How is this communication facilitated?
10. In terms of patient/caregiver education, who prepared it? Who delivers it? What information is provided and in what format? When are patients repatriated back to the referring centre? How is this communication facilitated?
11. Is the risk of CRS/ICANS predicted in any way before treatment? And does this change how care is delivered?
12. What does inpatient vs. outpatient monitoring look like for your bispecific DLBCL patients? What is measured? Where? By whom? How often? And for how long?
13. Have you implemented any practices that facilitate outpatient administration or monitoring? (e.g., prophylactic tocilizumab, patient check ins, standing orders in case of CRS/ICANS upon discharge, home health, oral steroids, etc.)
14. In the case of CRS/ICANS in an outpatient setting, how would patients be identified, triaged, and readmitted if necessary? Who is involved (e.g., on call staff, residents, day clinics)?
15. What are three critical success factors for bispecific implementation for DLBCL?
16. How would you like to see models of care evolve to support safe and efficient DLBCL implementation in the future?
17. Do you have any supportive tools you would be willing to share that we could adapt/include as a template/example in the publication?
18. How can experienced centres support other centres looking to administer bispecifics for DLBCL?

### Appendix A.2

**Table A2:** Examples of care paths in Canada and the envisioned evolution.

Example 1 (early adoption): referring centre does not administer BsAbs, all treatment occurs at an inpatient referral centre.
Example 2 (current progression): referring centre sends patient to inpatient referral centre for SUD and then delivers maintenance doses in their outpatient clinic, escalating AEs to local ER/ICU if needed.
Example 3 (future evolution): patients receive all treatment, including SUD, in an outpatient clinic and are admitted to the inpatient Internal Medicine ward of the affiliated hospital for a brief period of monitoring after each high-risk step-up dose.
Example 4 (future evolution): rural referral centre provides SUD and maintenance to all patients and escalates care to the local ER in case of CRS.
